# COVID-19 Contagion Risk Estimation Model for Indoor Environments

**DOI:** 10.3390/s22197668

**Published:** 2022-10-09

**Authors:** Sandra Costanzo, Alexandra Flores

**Affiliations:** 1 DIMES, Università della Calabria, 87036 Rende, Italy; 2 CNR-IREA Consiglio Nazionale delle Ricerche, 80124 Naples, Italy; 3 ICEmB, Inter-University National Research Center on Interactions between Electromagnetic Fields and Biosystems, 16145 Genoa, Italy; 4 CNIT, Consorzio Nazionale Interuniversitario per le Telecomunicazioni, 43124 Parma, Italy

**Keywords:** COVID-19, SARS-CoV-2, smart healthcare, contagion-risk monitoring, aerosol

## Abstract

COVID-19 is an infectious disease mainly transmitted through aerosol particles. Physical distancing can significantly reduce airborne transmission at a short range, but it is not a sufficient measure to avoid contagion. In recent months, health authorities have identified indoor spaces as possible sources of infection, mainly due to poor ventilation, making it necessary to take measures to improve indoor air quality. In this work, an accurate model for COVID-19 contagion risk estimation based on the Wells–Riley probabilistic approach for indoor environments is proposed and implemented as an Android mobile App. The implemented algorithm takes into account all relevant parameters, such as environmental conditions, age, kind of activities, and ventilation conditions, influencing the risk of contagion to provide the real-time probability of contagion with respect to the permanence time, the maximum allowed number of people for the specified area, the expected number of COVID-19 cases, and the required number of Air Changes per Hour. Alerts are provided to the user in the case of a high probability of contagion and CO_2_ concentration. Additionally, the app exploits a Bluetooth signal to estimate the distance to other devices, allowing the regulation of social distance between people. The results from the application of the model are provided and discussed for different scenarios, such as offices, restaurants, classrooms, and libraries, thus proving the effectiveness of the proposed tool, helping to reduce the spread of the virus still affecting the world population.

## 1. Introduction

The rapid worldwide spread of COVID-19 greatly impacts the entire population due to the increase in the mortality rate. The infection caused by COVID-19 disease is transmitted through inhalation or contact with very small droplets produced by coughing, sneezing, or talking. These particles cause a respiratory illness ranging from mild to moderate, which can dangerously transform into pneumonia [1]. Aerosol transmission is the dominant propagation factor, together with the large number of viral loads in the respiratory fluids [2].

Studies carried out in [3] identify three scenarios in which airborne transmission can occur between infectious people and those susceptible to contracting the infection, namely:

(a) People are very close to each other (<1–2 m), leading to the so-called “short-range aerial transmission”, which is effectively mitigated by physical distancing;
(b) People are sharing air in the same room, which is identified as “airborne shared space”;
(c) People do not share a room, or they are in very large rooms, leading to “long-distance airborne transmission”.

Recent studies are focused on the identification of factors potentially causing the increase in COVID-19 infections and their fatality rate. Various research papers are specifically finalized to identify the modalities through which the particulate material (PM) generates the wide spread of COVID-19. In particular, the study carried out in [4] shows that a possible early indicator of COVID-19 can be advised in conditions of atmospheric stability and high concentrations of PM; however, still today, the viability of the virus in PM has not been demonstrated, both in external as well as in internal environments.

The authors of [5] indicate various engineering controls and recommendations to maximize the protection of the population against the airborne spread of SARS-CoV-2 and any other small airborne virus-containing microdroplets, focusing on indoor environments. The authors discuss various options to be implemented for reducing infection in closed environments, such as the following ones: ventilation should be recognized as a means to reduce airborne transmission, ventilation rates should be increased by system modifications, air recirculation should be avoided, air cleaning and disinfection devices should be adopted as beneficial, and the number of people within the same indoor environment in epidemic situations should be minimized.

The study in [6] summarizes the environmental factors involved in the transmission of SARS-CoV-2, including a strategy to prevent the transmission of SARS-CoV-2 in internal environments, showing that the virus can persist on the surfaces of fomites for at least 3 days depending on the following environmental conditions: humidity and temperature. It is further indicated that the virus is stable for at least several hours if it is intentionally aerosolized and rapidly inactivated on surfaces with sunlight or high temperatures.

The authors of [2] propose two parameters as indicators of infection risk by COVID-19 spread, realizing a combination of the key factors that mainly control the transmission of diseases transmitted by air indoors, namely: the generation rate of aerosols containing viruses, respiratory flow rate, masking and its quality, ventilation rates and air cleaning, number of occupants, and duration of exposure. Recommended parameter values to minimize COVID-19 infection risk are finally provided in [2] for indoor environments.

All existing studies in the literature provide useful tools for risk estimation, including contact-tracing approaches based on Bluetooth Low Energy (BLE) [7,8]. Recently, an interesting method to assess indoor airborne transmission of COVID-19 has been also proposed [9], aiming to provide indoor safety guidelines that are able to identify a specific limit in the allowed amount of time for a person to spend in a given place. 

Around the world, there are ongoing efforts by scientists and governments to study the prevention and control of COVID-19. To this end, researchers have focused on evaluating pandemic prevention strategies [10,11]; other researchers have concentrated their studies on the Internet of Things (IoT) and smart sensor systems [12,13], which can collect and analyze data, as well as carrying out monitoring and warning tasks. Some studies concentrate on the application of big data to macroscopically address the COVID-19 disease [14]. Moreover, the use of Machine Learning, which is thought to greatly contribute to overall COVID-19 prevention and control [15,16], has caught the attention of a number of researchers. 

From the aforementioned literature review, we concluded that indoor air pollution has grown to be a significant issue, and its impacts can have serious health consequences, particularly in the wake of the COVID-19 pandemic. Therefore, it is essential to be aware of the risks to which we may be subject in order to act quickly when necessary. Mobile phone applications [17,18,19] have been at the forefront of tackling this problem in recent years, and the potential of mobile technology as a strategic support tool to restrict the spread of the pandemic played a very important role. Contact tracing using mobile apps is one such strategy, which can be carried out by local health workers physically or by using technology such as RFID [20] and wearable tracking devices [21]. Mobile apps for contact tracing can be used to track people who are contagious and to determine whether users are close to one another. These applications might undoubtedly reduce the spread of disease among people. Additionally, these tools let medical professionals recognize those who are at risk of contracting an infection, enabling early isolation and treatment to reduce both the intensity of the contagion and its transmission. 

In the present work, the analysis reported in the existing studies is considered as starting reference to implement a practical application for the accurate prediction of COVID-19 contagion risk in indoor environments. Specifically, an aerosol infection risk model based on the Wells–Riley equation [22] is adopted and the most frequent and dangerous situations are considered to derive the CO_2_ concentration to determine the air quality and to compute an indicator that allows maintaining social distance by measuring the Bluetooth RSSI (Received Signal Strength Indicator) relative to cell phones. The implemented application allows the evaluation of the influence of all parameters affecting indoor COVID-19 contagion risk, thus giving a tool for self-monitoring and controlling prevention measures.

As a specific novelty of the work, an indicator is included that allows controlling and maintaining social distance through the measurement of the Bluetooth RSSI (Received Signal Strength Indicator) relative to cell phones, demonstrating the feasibility of using Bluetooth RSSI in order to estimate the proximity between individuals and assess accuracy in various real-world scenarios. The implemented application allows the evaluation of the influence of all parameters affecting indoor COVID-19 contagion risk, featuring social distancing that is absent from comparable works, thus giving a tool for self-monitoring and controlling prevention measures.

The paper is organized as follows: Section 2 describes the adopted infection risk model and the modality used to obtain each involved parameter for the risk computation. The architecture of the proposed application is presented in Section 3, while results coming from the implementation of the application are discussed in Section 4. Finally, the conclusions are outlined in Section 5.

## 2. Model for Infection Risk by Aerosol

The aerosol infection risk model adopted in this paper is based on the Wells–Riley equation [22]. The purpose is the estimation of the probability of infection and the ventilation rate for most frequented indoor environments, such as offices, classrooms, restaurants, libraries, etc. The generated viral load is expressed in terms of the emission rate of quanta (q, quanta/hour). A quantum is defined as the dose of droplet nuclei in the air required to cause infection in 63% of susceptible individuals [22]. The Wells–Riley model [22] relates the probability of infection (*p*) with the number of inhaled quanta (*n*), according to the following equation:

(1) $$p=1-e^{-n}$$

Inhaled quanta n have a dependence on the concentration of quanta averaged over time (Cavg, quanta/m^3^), the volumetric respiratory rate of an occupant (Q_b_, m^3^/h), and the duration of the occupation (D, h), namely:

(2) $$n=C_{avg}\cdot Q_{b}\cdot D$$

The quantum concentration in air increases with time, from an initial value equal to zero, by following a “one minus exponential” form, which is the standard dynamic response of a fully mixed interior volume to a constant input source. A completely mixed material balance model can be applied to a room to calculate the concentration as:

(3) $$\frac{dC}{dt}=\frac{q}{V}-\lambda C$$

where:

- *q* is the quanta emission rate, also represented as ERq (quanta/h);
- *V* is the room volume (m^3^);
- *λ* is the first-order virus infectivity loss rate coefficient for quanta/h, due to:
  - Added effects of ventilation (λv, 1/h);
  - Deposition on surfaces (λdep, 1/h);
  - Virus disintegration (k, 1/h);
  - Additional control measures (λ_ACH, 1/h).
- *C* is the time-dependent airborne concentration of infectious quanta (quanta/m^3^).

Assuming the quantum concentration is equal to 0 at the beginning of the occupation, Equation (3) is solved, and the average concentration is determined as follows:

(4) $$C\left(t\right)=\frac{q}{\lambda V}\;\left(1-e^{-\lambda t}\right)$$

(5) $$C_{avg}=\frac{1}{D}\int_{0}^{D}C\left(t\right)\;dt=\frac{q}{\lambda V}\left[1-\frac{1}{\lambda D}\left(1-e^{\lambda D}\right)\right]$$

where D is the duration of exposure (hours).

Equation (5) can be rearranged to solve for the emission rate, *q*, namely:

(6) $$q=\lambda V\;C_{avg}*{\left[1-\frac{1}{\lambda D}\left(1-e^{-\lambda D}\right)\right]}^{-1}$$

The Wells–Riley equation can be also expressed as follows, according to [23]:

(7) $$p=\frac{N_{ca}}{N_{sus}}=1-e^{-l*q*Q_{b}*D/Q}$$

where:

- *p* = probability of infection risk;
- *Nca* = number of cases to develop infection;
- *Nsus* = number of susceptible people;
- *l* = number of source patients (infector);
- *Qb* = pulmonary ventilation rate of each susceptible person per hour (m^3^/h);
- *Q* = room ventilation rate (m^3^/h);
- *q* = quantum generation rate produced by one infector, also represented as ERq (quantum/h);
- *D* = exposure time (h).

In the following subsections, the key parameters for the analysis of the contagion risk and the application of the outlined model are described in detail.

### 2.1. Quanta Emission Rates for SARS-COV-2

With reference to quanta emission rates, the values recommended by the authors in [24,25] and reported in Table 1 are adopted in the present work. Values are displayed for four different activity levels, namely resting, light intensity, moderate intensity, and high intensity.

The estimation of the quantum emission rate of a person is carried out based on the respiratory parameters, the viral load, and the concentration of droplets expelled when carrying out different daily activities. The quanta emission rate (*q*, quanta h^−1^) is determined as:

(8) $$ER_{q}=C_{v}*V_{br}*N_{br}*\int_{0}^{10um}N_{d}\left(D\right)*dV_{d}\left(D\right)$$

where $C_{v}$ is the viral load in the sputum (RNA copies mL^−1^), $V_{br}$ is the volume of exhaled air per breath (cm^3^), $N_{br}\;$is the breathing rate (breath h^−1^), *N_d_* is the droplet number concentration (part. cm^3^), and $V_{d}\left(D\right)$ is the volume of a single droplet (mL) as a function of the droplet diameter (*D*). 

The study considers the case of natural rhythm respiration, with inspiration through the nose and expiration through the mouth and a size distribution for the particles given as: D_1_ = 0.8 µm, D_2_ = 1.8 µm, D_3_ = 3.5 µm, and D_4_ = 5.5 µm. The above diameters were used to calculate the corresponding volume of the droplets. With the results achieved in [24], Equation (8) can be simplified as:

(9) $$ER_{qj}=c_{v}*c_{i}*IR*\overset{4}{\underset{i=1}{\sum }}\left(N_{ij}*V_{i}\right)$$

where *j* indicates the different expiratory activities considered, and IR (m^3^ h^−1^) is the inhalation rate, given by the product of the breathing rate $N_{br}$ and the volume $V_{br}$, which is a function of the activity level of the infected subject.

### 2.2. Inhalation (Breathing) Rates

The breathing rate is quite an important parameter. Due to the activity performed by the individuals, a higher emission rate of quanta is produced. For example, when speaking, there is an increase in the emission of quanta, greater than the amount occurring with the respiratory rate, as more particles are emitted. For the present work, the parameters of the Exposure Factors Manual (Chapter 6 of the United States Environmental Protection Agency [26]) are used. In particular, data on the average daily inhalation rates for short exposures are presented in Table 2, which are related to age and activity level. As an example, data are only presented for some specific cases.

### 2.3. Mask Efficiency to Reduce Virus Emission

The efficiency evaluation for the different types of masks is based on literature [26,27,28], and it is reported in Table 3. 

### 2.4. Ventilation Rates 

The ventilation rate of an indoor environment is a key parameter to obtain a proper estimation of the infection rate due to aerosols. This parameter refers only to the replacement of indoor with outdoor air. As indicated in [26], a ventilation rate equal to 1 h does not mean that 100% of air is replaced in 1 h. The indoor air is displaced by the new air, but due to mixing, it does not work that way. Ventilation changes in different environments, and these changes are very important. For the analysis performed in this paper, the parameter Air Changes per Hour (ACH) is adopted, as can be observed from the calculation made in Table 4, where it is indicated that: if a certain place has a ventilation rate with outdoor air equal to 1 h, it means that, in 1 h, 63% of the indoor air is replaced by outdoor air; in 2 h, it equals to 86%, and in 3 h, the value is equal to 95%. If the ventilation rate is equal to 6 h^−1^, 97% of the air is replaced in 1 h by outside air. This justifies why the ventilation rate is very important for determining how long virus-laden aerosols can remain in a given space.

For the analysis conducted in the present work, the values in Table 5 are used, while an example is shown in Table 6 for the case of a classroom.

### 2.5. Decay Rate of Virus Infectivity in Aerosol

To compute the decay rate of the virus infectivity in aerosol, the values of relative humidity (*RH*), ambient temperature (*T*), and ultraviolet rays (*UV*) are considered [29], as reported in the following equation:

(10) $$\begin{array}{c} K_{infectivity}=0.16030+0.04018\left(\frac{T-20.615}{10.585}\right)+0.02176\left(\frac{RH-45.235}{28.665}\right)\\ +0.14369\left(\frac{S-0.95}{0.95}\right)+0.02636\left(\frac{T-20.615}{10.585}\right)\left(\frac{S-0.95}{0.95}\right) \end{array}$$

where:

- K_infectivity_ = decay constant for viral infectivity;
- T = temperature (°C);
- RH = relative humidity (%);
- S = integrated UV irradiance (W/m^2^), ranging from 0 (indoors) up to 10 (full sun noon).

According to the literature [3,26], values between 1 to 5um are considered for the size range of involved infectious particles.

### 2.6. Virus Removal Rate Using Controlled Systems

According to literature [28], for a portable HEPA (High-Efficiency Particulate Air) filter unit, the parameters shown in Table 7 are used. 

The air recirculating through an Heating, Ventilating, Air Conditioning (HVAC) system can present particle leaks, so it is important to put filters in these systems to increase the elimination of said particles. Table 8 shows an example to calculate Air Changes Per Hour (ACH) for additional control measures, using data taken from Table 9 and based on [30].

### 2.7. Disease Prevalence in a Certain Area

The disease prevalence factor depends on the state of the pandemic in each region and period of time, as well as the dynamics of the disease and its infectivity in different types of cases, which are not exactly known. In the case of Italy, the online tool available at the link https://lab24.ilsole24ore.com/coronavirus/ (accessed on 4 August 2022) is adopted to find the current estimated fraction of infectious people in a specific region or province. The calculation of this parameter is difficult to quantify with precision, but the correct order of magnitude can be obtained on the basis of data relative to the prevalence of the disease and the epidemiological models. A person is considered contagious in the week around the onset of the first symptoms. In the same way, there is also a fraction of the population with asymptomatic people who could increase infections. These parameters must be also taken into account in the estimation stage.

Table 10 shows an estimate for the province of Cosenza in Italy, with reference to the month of May 2021. 

### 2.8. Fraction of Immune People

Immunity can be acquired due to vaccination or due to disease. The fraction of vaccinated people can be obtained from web sources, such as the New York Times vaccine tracker, or in the case of Italy and the other countries, at the following link: https://lab24.ilsole24ore.com/coronavirus/ (accessed on 4 August 2022). The effectiveness percentage of the main vaccines against COVID-19 are detailed in [31].

The number of immune people is equal to the product of the number of vaccinated people and the effectiveness of the vaccine, namely:

(11) $$N_{in}=N_{vac}*V_{eff}$$

where:

- *N_in_* = the number of immune people;
- *N_vac_* = the number of people vaccinated;
- *V_eff_* = percentage of vaccine efficacy.

As the disease progresses, the population acquires immunity, which may be higher than 20% in some countries and minimizes the number of people who could become infected. For Italy and other countries, the number of infected people can be obtained in real-time at the following link: https://lab24.ilsole24ore.com/coronavirus/ (accessed on 4 August 2022).

In Italy, 4,146,722 people had been infected before May 2021, that is, 6.87% of the population.

### 2.9. CO_2_ Emission Rate

CO_2_ emission rates can be computed by applying the following rules:

1. Define the CO_2_ generation rate according to parameters of age, sex, and metabolic rate (see Table 11);
2. Compute the metabolic rate (met) according to the activity being carried out (see Table 12);
3. In case the value selected in Table 12 is > 4, the highest value in Table 11 should be used;
4. Use the obtained value to perform the computations in Equations (12) and (13) (CO_2_ emission rate and average CO_2_ mixing ratio, respectively).

On the basis of the outlined rules and tabulated data, the CO_2_ emission rate can be computed as:

(12) $$CO_{2}=\left(met*N\right)*\frac{1}{Pr}*\frac{273.15+T}{273.15}$$

where:

- *CO_2_* = emission rate (all people), L/s;
- *Pr* = pressure, atm;
- *met* = generation rate, L/s;
- *N* = number of people present;
- *T* = temperature, °C.

In addition, the CO_2_ mixing ratio is given by the formula:

(13) $$CO_{2}{}_{AVG\_m}=\frac{\left(CO2*3.6\right)}{\lambda v\;V}*\left(1-\left(\frac{1}{\lambda v\;D}\right)*\left(1-e^{-\lambda v\;D}\right)\right)*1,000,000+B_{CO_{2}}$$

where:

- $CO_{2}{}_{AVG\_m}$ = avg mixing ratio (ppm);
- *CO_2_* = emission rate (all persons) (L/s);
- *λv* = added effects of ventilation (1/hours);
- *D* = duration of exposure (hours);
- $B_{CO_{2}}$= background CO_2_ outdoors (ppm), (350 to 450 ppm range).

## 3. Application Design

**Figure 1:**
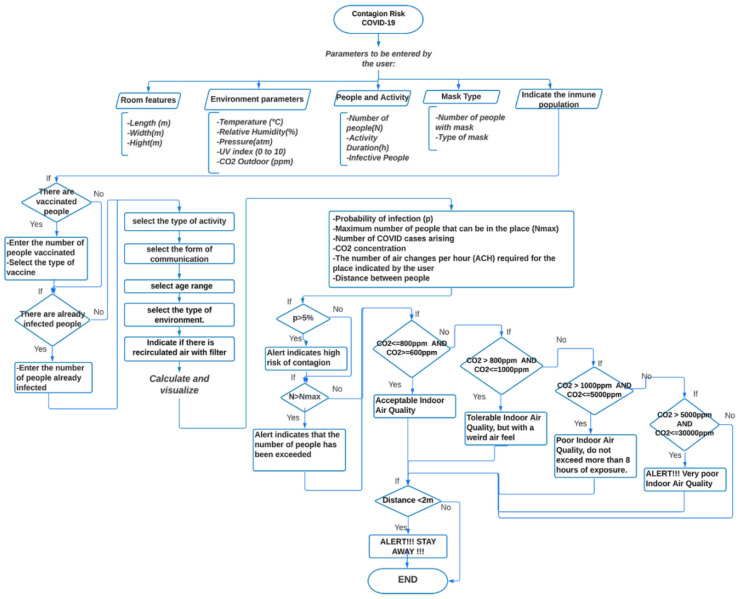
Flow-chart of the application for COVID-19 contagion risk estimation.

The outlined model for COVID-19 contagion risk estimation is adopted to design a specific application in Android Studio. The schematic diagram is illustrated in Figure 1. In particular, user-entered parameters are expected, such as room and environment characteristics, number of people present, permanence time, number of people using masks, and type of mask. To identify the immune people, the user is asked also to enter the number of vaccinated people and the number of people who have already been infected with COVID-19. Information relative to the activity, age range, and location scenario (domestic, schools, food service, hotels resorts/dormitories, office buildings, public assembly spaces, sports, and entertainment) must be further provided. Based on the above parameters, the application provides data regarding the probability of contagion with respect to permanence time, the maximum allowed number of people for the specified area, the expected number of COVID-19 cases, the required number of Air Changes per Hour (ACH), and an indicator of infection based on air quality through CO_2_ concentration. If achieving high values for the probability of contagion and the CO_2_ concentration, the application displays an alert to the user. Additionally, a proximity meter is incorporated, through the RSSI, by exploiting the strength of the Bluetooth signal associated with the devices of the present people, which allows for obtaining a distance estimate. The higher the RSSI value, the stronger the signal, which corresponds to a reduced distance between devices. If the distance between people is less than two meters, the application displays an alert.

**Figure 2:**
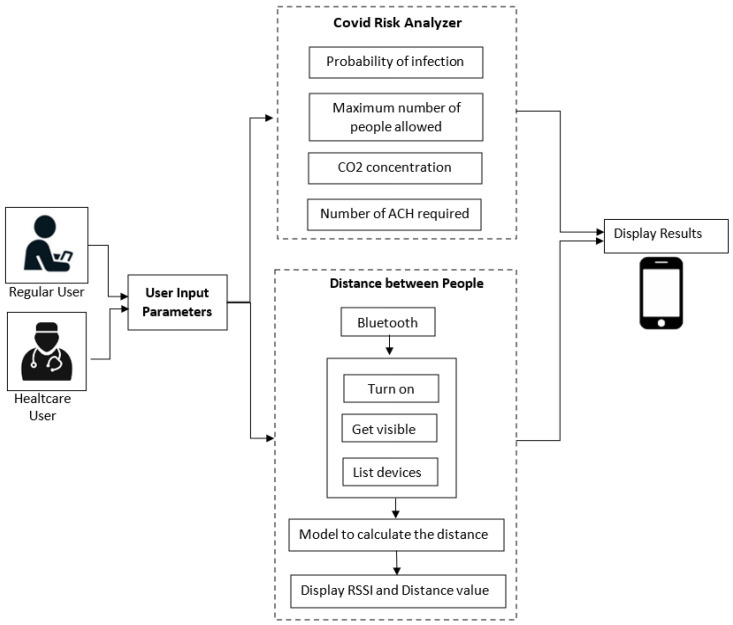
Application architecture.

A detailed description of the architecture relative to the proposed system is shown in Figure 2. It is represented by an Android application using JAVA as a programming language. The system initially allows selecting between two types of users: regular users and healthcare users. Then, there are three individual blocks, namely the user input parameters, the risk analyzer, and the measuring distance between people. They are described in detail as follows:

(a) User input parameters:

In this block, the application asks the user to enter some parameters related to the prediction of COVID according to the specific indoor environment. In the case of a regular user, an interface is presented where he can enter known parameters and easily understand and efficiently navigate the application. In this case, some parameters are omitted, such as the dimensions of the place (standard dimensions are considered for each environment), the number of infected people, the number of vaccinated people, the number of people already infected, and the vaccine type. On the other hand, in the case of the healthcare user, he is asked to enter these more specific parameters to obtain more detailed results.
Data collected through this module are sent to the COVID risk analyzer block.

(b) COVID Risk Analyzer:

The risk analyzer takes the input from the previous module. It evaluates the received data and computes the probability of COVID infection, the maximum number of people allowed in the place, the concentration of CO_2_, and the number of ACH required in that environment.
In addition, this block allows sending some alert notifications to the user under the following conditions:
  - The probability of contagion is high;
  - The maximum number of people allowed in the place is exceeded;
  - The air quality is not good, that is, when the CO_2_ level is greater than 1000 ppm.

(c) Distance between people

This block collects Bluetooth data, including the estimated RSSI value, along with the distance between nearby devices. To achieve this purpose, the user must activate his Bluetooth. If the distance between users is less than two meters, the user is provided with an alert notification.

Various models exist in the literature for the estimation of distance [32,33]. In the present work, a linear approximation model is adopted by specifically applying the following expression:

(14) y=a1∗sin(b1∗x+c1)+a2∗sin(b2∗x+c2)+a3∗sin(b3∗x+c3)

where:

- y = distance (meters);
- x = RSSI (dBm);
- a1 = 268.5; b1 = 0.4437; c1 = 2.702; a2 = 189.7; b2 = 0.5509; c2 = −0.8691; a3 = 1.633; b3=2.182; c3 = −3.434.

**Figure 3:**
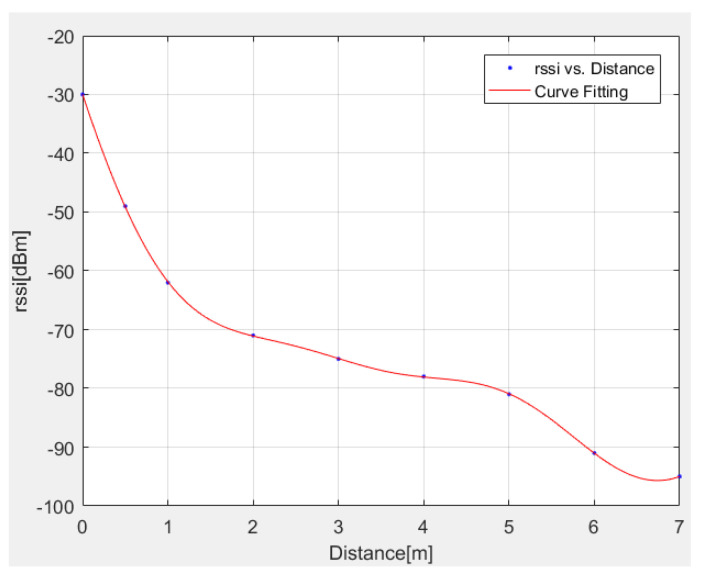
Comparison between exact and interpolated (Equation (14)) RSSI data.

The above interpolation model is successfully compared in Figure 3 with exact RSSI data.

## 4. Results

**Figure 4:**
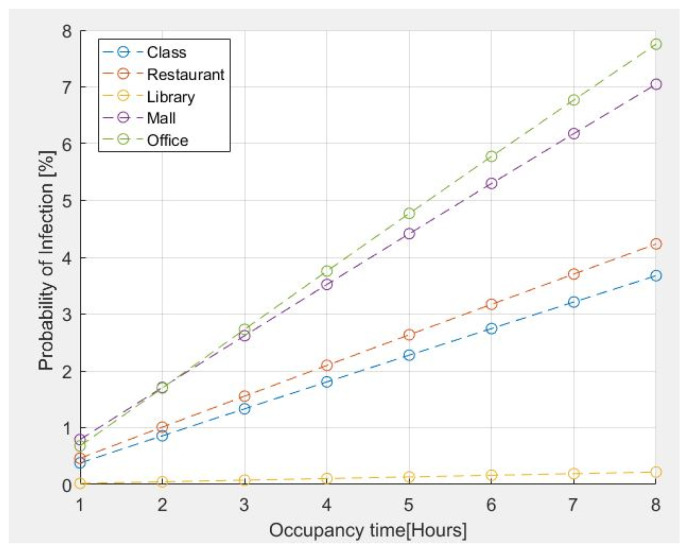
Probability of infection for some common environments vs. occupancy time.

The results for the probability of infection within certain common environments are shown in Figure 4. For the above analysis, the presence of an infected person is assumed in all simulated environments. The emission rates of quanta adopted for the different activities are those reported in Table 1, specifically detailed as follows:

- 11.4 quanta/h for a Classroom–Light Intensity–Speaking;
- 26.3 quanta/h for a Restaurant–Moderate Intensity-Speaking;
- 2 quanta/h for a Library–Sedentary/Passive-Oral breathing;
- 63.1 quanta/h for a MALL–High Intensity-Speaking;
- 11.4 quanta/h for an Office–Light Intensity-Speaking.

The number of people present is assumed as follows:

- Classroom = 42 people;
- Restaurant = 280 people;
- Library =20 people;
- Mall = 400 people;
- Office = 10 people.

The assumed age range for all cases is from 21 years up to 31 years.

The risk estimation reported in Figure 4 indicates that the probability of contagion for an exposure time of two hours is less than 2% for classrooms, restaurants, and libraries. However, tends to increase if the stay time exceeds 2 h in the case of an office of 100 m^2^ for 10 people because it is a small place. The probability of contagion is high if the permanence time is greater than 1h30; furthermore, in the case of a shopping center, the probability of contagion is greater due to the presence of a large number of people who move and speak loudly, even though it is a large space.

**Figure 5:**
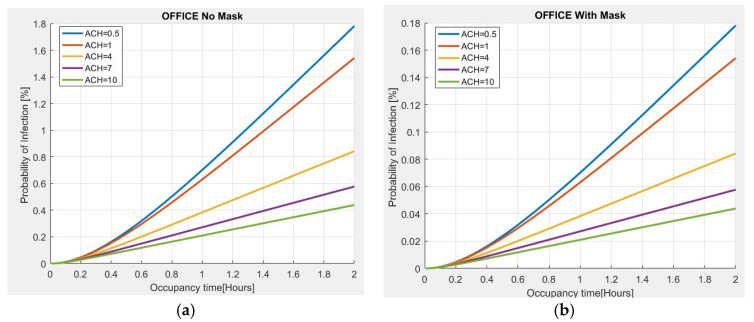
Probability of infection vs. permanence time for different ACH values in an office: (**a**) without mask; (**b**) with mask.

As further validation of the proposed model, an estimate of the probability of infection, with respect to the residence time and the number of Air Changes per Hour (ACH), is made for a 100 m^2^ office, 3 m high, with 10 people present. The evaluation carried out in Figure 5a shows that if an asymptomatic person infected with COVID-19 is present in the place and no person wears a mask for a stay time of 2 h, the probability of contagion is approximately 2% for the minimum value of ACH = 0.5, observing that the risk of infection increases with the passage of time. However, if the ACH increases to 4, the risk of infection tends to decrease to approximately 0.8%; with an ACH = 10, the probability of infection is less than 0.5%, thus indicating the importance of ventilation in indoor environments to reduce the probability of infection. The use of a mask is also very important because the probability of infection is significantly reduced as compared to the previous case (Figure 5b).

**Figure 6:**
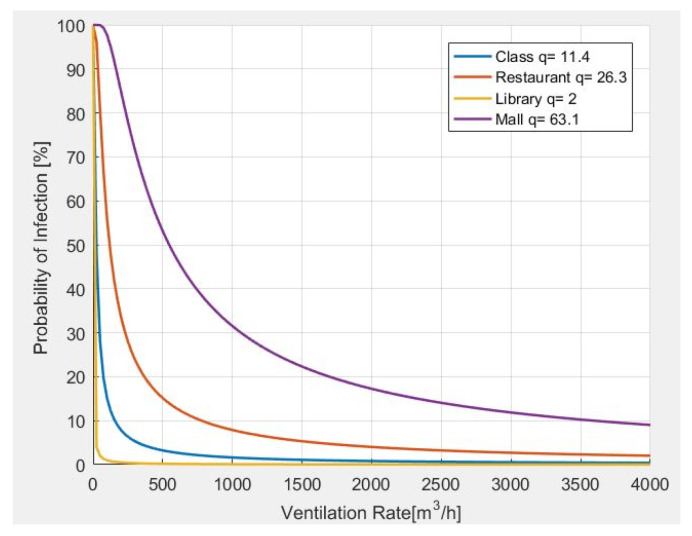
Probability of infection and required ventilation rate for different indoor environments.

In Figure 6, the results from the analysis of the probability of infection, as related to the ventilation rate, are carried out. In particular, it is observed that the greater the number of quanta generated by a person according to the carried activity, the more ventilation is required. 

**Figure 7:**
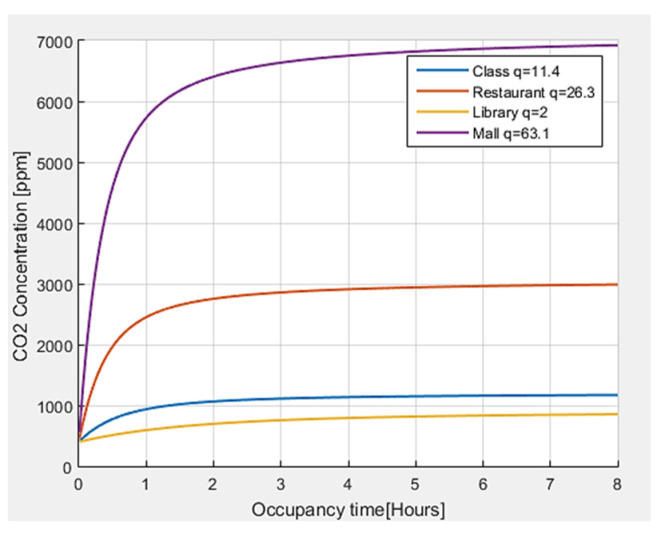
CO_2_ concentration with respect to permanence time for different indoor environments.

Finally, an analysis of the CO_2_ concentration is carried out for different environments, obtaining the results shown in Figure 7. It is observed that the greater the number of quanta required by a person in a closed environment, the greater the CO_2_ concentration. In the same way, according to the activity carried out and the number of people present, the levels of CO_2_ tend to grow, and they tend to significantly increase with time.

The threshold values defined in the literature [34] for the concentration of CO_2_ have been taken as references. For outdoor air, a CO_2_ concentration <450 ppm is generally shown, and the reference value of CO_2_ for indoor environments in pandemic situations is equal to 800 ppm, although values up to 1000 ppm are also considered acceptable. In the case of CO_2_, with values higher than 1000 ppm, the exposure time should not exceed 8 h. When CO_2_ levels constantly increase over time, it is an indicator that ventilation is not adequate for the number of occupants and the carried-out activity.

To verify the effectiveness of the proposed application, a comparison is made with the Airbone.cam platform [19]. For all considered environments, the following parameters are taken as reference: the number of people present = 20, duration of the activity = 2 h, and age of the people present range from 21 to 31 years old. In addition, all people are assumed to wear surgical masks. In Table 13, the comparison results are displayed between our proposed application and that developed in [19], with an average relative error of 10.65%.

The RSSI value of Bluetooth and Wi-Fi must first be obtained in order to determine the relative distance between two phones. With this value, it is simple to determine the distance and the accuracy. Table 14 shows a comparison between real and estimated distance data over Bluetooth and Wi-Fi, observing an average error of 2.47% for the case of Bluetooth and 4.22% for the case of Wi-Fi. According to the obtained experimental results, the accuracy of Bluetooth and Wi-Fi can be fixed to 7 m. 

A further comparison in terms of accuracy, relative error, and battery consumption is performed with respect to reference work [8], and the relative findings are displayed in Table 15. These results demonstrate that the Bluetooth method is an effective and efficient way to obtain the distance value in an indoor environment, considering accuracy and power consumption. Additionally, as compared to [8], our proposed method offers improved distance precision and a reduced error rate.

**Figure 8:**
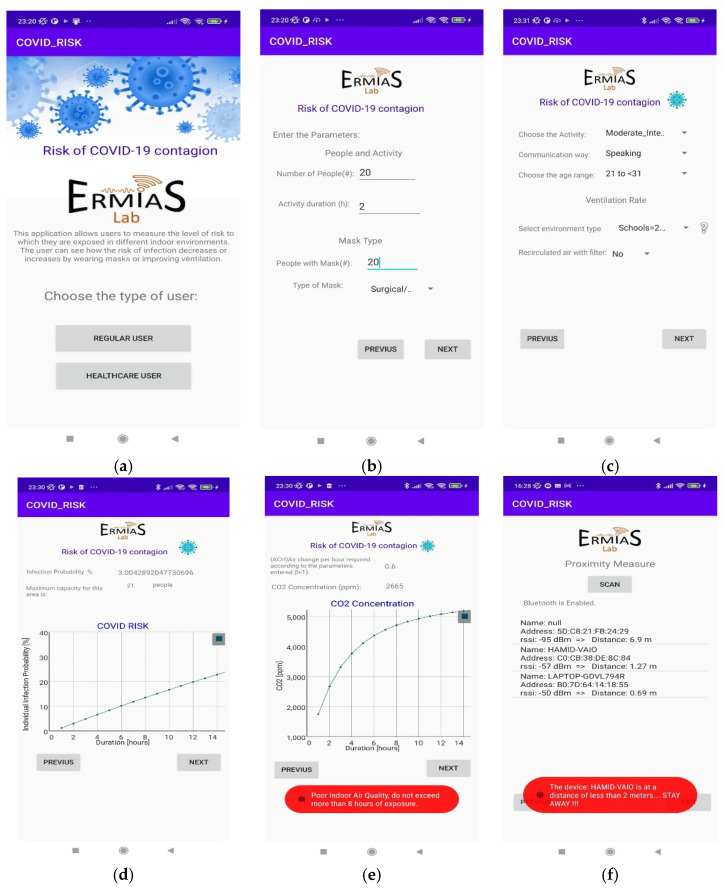
Android application results: (**a**) main screen of the application; (**b**) entry of parameters related to the type of people; (**c**) entry of parameters related to the type of environment; (**d**) probability of infection with respect to permanence time and the maximum number of allowed people; (**e**) required Air Changes per Hour (ACH) and concentration of CO_2_; (**f**) proximity meter.

In Figure 8, the screenshots of Android application reporting: main screen of the application (Figure 8a); entry of parameters related to the type of people (Figure 8b); entry of parameters related to the type of environment (Figure 8c); the infection probability vs. permanence time and the maximum number of people allowed (Figure 8d); the required Air Changes per Hour (ACH) and the CO_2_ concentration vs. duration (Figure 8e); and the estimation of distance (proximity meter) with relative alert (Figure 8f) are reported.

## 5. Conclusions

An accurate model, based on Wells–Riley probabilistic approach for interior spaces, has been presented and accurately outlined in this work for the prediction of COVID-19 contagion risk. The proposed model has been implemented in a specific Android application for real-time monitoring of COVID-19 risk in indoor environments. All relevant parameters affecting aerosol transmission, such as quanta emission rate, inhalation rate, permanence time, and ventilation rate, are properly included in the model to derive the probability of infection, thus leading to the implementation of practices to avoid or minimize the risk. The proposed interactive tool can be usefully applied for real-time monitoring of indoor, largely frequented environments, such as offices, restaurants, and classrooms, thus usefully helping to mitigate the risk due to aerosol diffusion of COVID-19.

## Figures and Tables

**Table 1:** Quanta emission rates for SARS-CoV-2 (quanta/hour).

Activity	Oral Breathing	Speaking	Aloud Speaking or Singing
Sedentary/Passive Resting	2	9.4	60.5
Light Intensity/Standing	2.3	11.4	65.1
Moderate Intensity	5.6	26.3	170
High Intensity	13.5	63.1	408

**Table 2:** Average daily inhalation rates for Short-Term Exposure Values.

Activity Level	Age Group (Years)	Mean (m^3^/min)	95th Percentile (m^3^/min)
Sedentary/passive	16 to <21	5.3 × 10^−3^	7.2 × 10^−3^
	21 to <31	4.2 × 10^−3^	6.5 × 10^−3^
	31 to <41	4.3 × 10^−3^	6.6 × 10^−3^
Light Intensity	16 to <21	1.2 × 10^−2^	1.6 × 10^−2^
	21 to <31	1.2 × 10^−2^	1.6 × 10^−2^
	31 to <41	1.2 × 10^−2^	1.6 × 10^−2^
Moderate Intensity	16 to <21	2.6 × 10^−2^	3.7 × 10^−2^
	21 to <31	2.6 × 10^−2^	3.8 × 10^−2^
	31 to <41	2.7 × 10^−2^	3.7 × 10^−2^
High Intensity	16 to <21	4.9 × 10^−2^	7.3 × 10^−2^
	21 to <31	5.0 × 10^−2^	7.6 × 10^−2^
	31 to <41	4.9 × 10^−2^	7.2 × 10^−2^

**Table 3:** Mask efficiency in reducing virus inhalation by a susceptible person.

Mask Type	Exhalation Mask Efficiency	Inhalation Mask Efficiency	Description
N95 masks (KN95, FF2)	90%	90%	These types of masks are the most recommended ones because if they are worn well, their efficiency for large particles with viruses is equal to 99% or more. In the present work, 90% efficiency is assumed because, in real life, a large part of the population does not wear masks correctly, so there may be particle leaks [26].
N95 masks that have an exhalation valve.	0%	0%	This type of mask makes most of the air escape through the valve, and there is no good filtering. These masks are good for occupational exposure if a worker is sanding, drilling, etc., but they do not protect against the particles that are exhaled [26].
Cloth, surgical	50%	30%	This value is applicable to the general population and considering the various ways in which they are worn [27].
Face shields worn without a mask.	23%	23%	The efficiency is low due to the limited inertia of the exhaled particles under normal conditions of respiration or conversation [28].

**Table 4:** Percentage of Air Changes per Hour.

	Percentage of Initial Air Remaining: $exp\;\left(-ACH\;*\;time_{\left[hours\right]}\right)*\;100\%\;$	Percentage of Indoor Air Replaced by Outdoor Air
After 1 h	exp (−1) ∗ 100% = 37%	63%
After 2 h	exp (−2) ∗ 100% = 14%	86%
After 3 h	exp (−3) ∗ 100% = 5%	95%

**Table 5:** Minimum Ventilation Rates in Breathing Zone.

	People Outdoor Air Rate (Rp)		Area Outdoor Air Rate (Ra)		Default Values			
					Occupant Density	Combined Outdoor Air Rate		
Occupancy Category	cmf/person	L/s. Person	cmf/ft^2^	L/s.m^2^	#/1000 ft^2^ or #/100 m^2^	cfm/person	L/s. person	Air class
Domestic	5	2.5	0.06	0.3	15	9	4.5	1
Schools	10	5	0.12	0.6	35	13	6.7	1
Food Service	7.5	3.8	0.18	0.9	70	10	5.1	2
Hotels Resorts/Dormitories	5	2.5	0.06	0.3	10	11	5.5	1
Office Buildings	5	2.5	0.06	0.3	10	11	5.5	1
Public Assembly Spaces	5	2.5	0.06	0.3	150	5	2.7	1
Sports and Entertainment	20	10	0.18	0.9	7	45	23	2

**Table 6:** Example of ventilation rate computation for the case of a classroom.

People outdoor air rate (Rp)	5	L/s.person	From Table 5
Area outdoor air rate (Ra)	0.6	L/s·m^2^	From Table 5
Occupant density (Nd)	35	Per/100 m^2^	From Table 5
Surface area (a)	100	m^2^	For a specific location
Height of room (h)	3	m	For a specific location
Volume of room (V)	300	m^3^	V = a ∗ h
Number of occupants (N)	35	People	N=Nd∗a/100
Vent. Rate	235	L/s	Vent Rate=(N∗Rp+a∗Ra)
Vent. in h^−1^	2.82	h^−1^	Vent. in 1 h =(Vent rate ∗ 36,000 ∗ 0.001/V[M1] )

**Table 7:** Virus removal rate for a portable HEPA filter.

HEPA flow rate	440	m^3^ h^−1^
Room size (Volume)	147	m^3^
Removal rate=(HEPA flow rate)/(Volume)	3.0	h^−1^g

**Table 8:** Air Changes Per Hour (ACH) for additional control measures.

Parameters	Values	Units	Description
Recirculated flow rate (Rfr)	300	m^3^/h	
Volume of room (V)	100	m^3^	
Filter efficiency (Feff)	20	%	Enter from Table 9
Removal in ducts, air handler (Rd)	10	%	Assuming some losses in bends, air handler surfaces, etc.
Other removal measures (Rot)	0	%	Germicidal UV (or other systems), from specs or the system
ACH for additional control measures	0.9	h^−1^	$ACH=\frac{\mathrm{Rfr}\;}{V}*MIN\left(F_{eff}+Rd+Rot\right)$

**Table 9:** Minimum Efficiency Reporting Value (MERV) Parameters.

Standard 52.2	Composite Average Particle Size Efficiency Size Range, (um)			
Minimum Efficiency Value (MERV)	Range 1	Range 2	Range 3	Average Arrestance, (%)
	(0.3–1.0)	(1.0–3.0)	(3.0–10.0)	
1	n/a	n/a	E3 < 20	Aavg < 65
2	n/a	n/a	E3 < 20	65 ≤ Aavg < 70
3	n/a	n/a	E3 < 20	70 ≤ Aavg < 75
4	n/a	n/a	E3 < 20	75 ≤ Aavg
5	n/a	n/a	20 ≤ E3	n/a
6	n/a	n/a	35 ≤ E3	n/a
7	n/a	n/a	50 ≤ E3	n/a
8	n/a	20 ≤ E2	70 ≤ E3	n/a
9	n/a	35 ≤ E2	75 ≤ E3	n/a
10	n/a	50 ≤ E2	80 ≤ E3	n/a
11	20 ≤ E1	65 ≤ E2	85 ≤ E3	n/a
12	35 ≤ E1	80 ≤ E2	90 ≤ E3	n/a

**Table 10:** Estimate of disease prevalence for the province of Cosenza in Italy (May 2021).

New cases per day Cosenza per 100,000 people (Nc)	106	
Fraction of asymptomatic or unreported cases (As)	50%	
Duration of infective period (Dip)	7	days
Fraction of population infective at a given time (N_inf)	1.48%	$N\_inf=Nc*\frac{0.00001}{1-As}*Dip$

**Table 11:** CO_2_ generation rates for ranges of ages and physical activity at 273 °K and 101 KPa.

			CO_2_ Generation Rate (L/s)						
Age (y)	Mean Body	BMR	Level of Physical Activity (met)						
	Mass (Kg)	(MJ/day)	1.0	1.2	1.4	1.6	2.0	3.0	4.0
**Males**									
16 to <21	77.3	7.77	0.0037	0.0045	0.0053	0.0060	0.0059	0.0113	0.0150
21 to <30	84.9	8.24	0.0039	0.0048	0.0056	0.0064	0.0063	0.0120	0.0160
30 to <40	87.0	7.83	0.0037	0.0046	0.0053	0,0061	0.0059	0.0114	0.0152
40 to <50	90.5	8.00	0.0038	0.0046	0.0054	0.0062	0.0060	0.0116	0.0155
**Females**									
16 to <21	65.9	6.12	0.0029	0.0036	0.0042	0.0047	0.0059	0.0089	0.0119
21 to <30	71.9	6.49	0.0031	0.0038	0.0044	0.0050	0.0063	0.0094	0.0126
30 to <40	74.8	6.08	0.0029	0.0035	0.0041	0,0047	0.0059	0.0088	0.0118
40 to <50	77.1	6.16	0.0029	0.0036	0.0042	0.0048	0.0060	0.0090	0.0119

**Table 12:** Values of physical activity levels (met).

Activity	M (met)	Range
Dancing—aerobic, general	7.3	
Health club exercise classes—general	5.0	
Kitchen activity—moderate effort	3.3	
Lying or sitting quietly		1.0 to 1.3
Sitting reading, writing, typing	1.3	
Sitting tasks, light effort (e.g., office work)	1.5	
Sitting quietly in religious service	1.3	
Sleeping	0.95	
Standing quietly	1.3	
Standing tasks, light effort (e.g., store clerk, filing)	3.0	
Walking, less than 2 mph, level surface, very slow	2.0	
Walking, 2.8 mph to 3.2 mph, level surface, moderate pace	3.5	

**Table 13:** Comparison results with reference [19].

Environment	Risk of Infection (%) My App	Risk of Infection (%) Airborne.cam	Relative Error (%)
Domestics	8.5	9.01	5.66
Schools	3.00	3.70	18.92
Food Service	1.05	0.97	8.25
Hotels Resorts/Dormitories	10.28	12.56	18.15
Office Buildings	12.76	10.90	17.06
Public Assembly Spaces	0.002	0.0019	5.26
Sports and Entertainment	0.78	0.79	1.27
**mean**			**10.65**

**Table 14:** Comparison results between real and estimated distance values measured with Bluetooth and Wi-Fi.

Real Distance (m)	Bluetooth Estimated Distance (m)	Bluetooth Relative Error (%)	Wi-Fi Estimated Distance (m)	Wi-Fi Relative Error (%)
0.5	0.5044	0.88	0.4951	0.98
1	0.9978	0.22	1.1442	14.42
2	1.8658	6.71	1.8177	9.11
3	3.1244	4.15	3.0719	2.40
4	4.1023	2.56	4.0710	1.77
5	4.8898	2.20	4.8755	2.49
6	6.1035	1.73	6.0883	1.47
7	6.9037	1.38	6.9236	1.09
**mean**		**2.47**		**4.22**

**Table 15:** Comparison between our method and the method used in [8].

	Accuracy	Relative Error	Power Consumption
Our Bluetooth method	7 m	2.47%	44 h
Bluetooth method of [8].	1.5 m	13.8%	41 h

## Data Availability

The data presented in this study are available on request from the corresponding author.
